# Middle-Aged and Older Caregivers’ Depressive Symptoms and Multiple Sources of Social Support

**DOI:** 10.1093/geroni/igaf122.3320

**Published:** 2025-12-31

**Authors:** Seungjong Cho, Yeon Jin Choi, Sun Kyung Kim

**Affiliations:** Texas Tech University, Lubbock, Texas, United States; University of Kentucky, Lexington, Kentucky, United States; Case Western Reserve University, Cleveland, Ohio, United States

## Abstract

Providing care in middle and later life can be physically and emotionally challenging, often contributing to heightened depressive symptoms among caregivers. Since depressive symptoms are associated with physical decline, higher mortality risk, and reduced quality of life, it is essential to identify protective factors that can mitigate this adverse effect. While previous research highlights the benefits of social support, most studies aggregate different sources rather than assessing their distinct effects. This study aims to compare the effects of four different sources of social support (spouse or partner, children, relatives, and friends) on depressive symptoms among middle-aged and older caregivers. We analyzed merged data from the 2020 Health and Retirement Study (N = 1,068). The results from multiple linear regression with multiple imputation indicated that only spousal support was a statistically significant predictor of depressive symptoms among caregivers when all social support variables were entered simultaneously. Specifically, the coefficient for spousal support was -.236 (p=.016), meaning that as spousal support increases by one unit, depressive symptoms decrease by an average of 0.236 units. Among the covariates, higher levels of depressive symptoms were also linked to younger age, being female (compared to male), and lower self-rated health. These findings underscore the critical role of spousal support in mitigating caregivers’ depressive symptoms. Given its unique significance, interventions should focus on strengthening spousal relationships through counseling, communication training, and caregiver support programs tailored for couples. Additionally, since younger caregivers exhibited higher depressive symptoms, targeted mental health resources and caregiver support services should prioritize these vulnerable groups.

